# Dataset of wet desulphurization scrubbing in a column packed with Mellapak 250.X

**DOI:** 10.1016/j.dib.2020.106383

**Published:** 2020-10-08

**Authors:** D. Flagiello, A. Erto, A. Lancia, F. Di Natale

**Affiliations:** Department of Chemical, Materials and Production Engineering, University of Naples Federico II, P.le Tecchio, 80 - 80125 Naples, Italy

**Keywords:** Sulfur dioxide (SO_2_), Flue-gas desulphurization (FGD), Exhaust gas cleaning system (ECGS), Absorption column, Wet-scrubbers, Packed tower, Structured packing

## Abstract

Flue-Gas Desulphurization (FGD) is a fundamental process commonly adopted for the treatment of exhausts deriving from both stationary and mobile sources. The removal of SO_2_ from flue gasses can be made through different technologies and absorption offers the highest versatility for a large spectrum of applications.

The data presented in this paper derive from FGD experiments carried out in a pilot wet scrubber equipped with a structured packing (Hastelloy C-22, Mellapak 250.X). The experiments aim to determine the SO_2_ removal efficiency from a simulated flue-gas in different operating conditions, similar to those observed in common wet FGD processes. Experimental data are reported in terms of gas velocity, concentration of SO_2_ in the flue-gas, liquid/gas feed ratio, fluids temperature and pressure. The dataset also includes the measurements of several working parameters, *i.e.* pressure drops in the column, wash water pH, relative humidity of the outlet gas and temperatures of gas and liquid flowing out of the FGD unit.

The collection of these data could be useful in future studies and in the analysis of FGD units, also to design/improve large-scale absorption columns with structured packing, using various scrubbing liquids and in different operating conditions.

## Specifications Table

**Table:** 

Subject	Fluid Flow and Transfer Processes
Specific subject area	Absorption processes for SO_2_ removal from flue-gas, *i.e.* wet Flue Gas Desulphurization (FGD).
Type of data	Figures and Tables.
How data were acquired	The data reported in this document were acquired in a pilot-scale scrubber by measuring: • SO_2_ gas concentrations with an ABB O2020® gas analyser, with a range of detection 0 - 5000 ppm_v_ and an accuracy of ±5 ppm_v_; • Gas temperatures with a HOBO® four channels digital thermometer (PCE T-390 model, with accuracy ±0.1 °C); • Gas pressure drops in column with a differential pressure gage (FLUKE Corporation, Air Flow Meter 922 model with accuracy of ±0.1 mm_H2O_); • Gas humidity with a HOBO® onset digital humidity controller (UX100–23 model with accuracy of ±0.1% of relative humidity); • Liquid temperatures with a WINGONEER™ mini digital LCD thermometer (with accuracy of ±0.1); • Liquid pH with a HOBO® digital pH-meter (PCE-228 model, with accuracy of ± 0.01).
Data format	Raw and Analysed data
Parameters for data collection	The datasets were collected during SO_2_ absorption experiments from simulated flue-gasses under different experimental conditions typical of FGD processes in packed towers, *i.e.* by testing: • Four flue-gas flow rates (28 – 40 m^3^⋅h ^−^ ^1^, which correspond to a flue-gas velocity in the range 1.00 – 1.41 m⋅s ^−^ ^1^); • Four liquid flow rates (40 – 130 L⋅h ^−^ ^1^, which allow to achieve different liquid-to-gas ratios, in the range 1.00 – 4.64 L⋅m ^−^ ^3^); • Three flue-gas temperatures (25 – 60 °C); • Five different scrubbing liquids (with pH values ranging from 3 to 9.4). On contrary, some parameters were kept as constant during the experiments: • Liquid temperature, which was set at 25 °C; • Inlet flue gas humidity, which was fixed at 13 – 25% relative value, in the temperature range 25 – 60 °C.
Description of data collection	During the scrubbing desulphurization process, the following experimental data were continuously acquired: • SO_2_ outlet concentration of the simulated flue-gas; • Outlet temperature of the simulated flue-gas; • Gas pressure drops in column; • Outlet relative humidity of the simulated flue-gas; • Outlet temperature of the scrubbing liquid; • pH of the outlet scrubbing liquid. The datasets reported in this work were collected when both fluid-dynamic and hydrodynamic steady-state conditions were reached in the column.
Data source location	Department of Chemical, Materials and Production Engineering of the University of Naples Federico II, P.le Tecchio, 80 - 80,125 Naples, Italy.
Data accessibility	Data are included in this article.
Related research article	D. Flagiello, F. Di Natale, A. Lancia, A. Erto, Characterization of mass transfer coefficients and pressure drops for packed towers with Mellapak 250.X, Chemical Engineering Research and Design 161 (2020) 340–356. https://doi.org/10.1016/j.cherd.2020.06.031

## Value of the Data

• The dataset can be used in future studies and analysis of flue-gas desulphurization units, and in the set-up and test of accurate models for the support of the design and the optimization of FGD units [1], [2], [3].
• These data could be useful for researchers and engineers that are committed in the design or operation improve of large-scale absorption columns equipped with structured packing with high separation-efficiency [1], [2], [3], [4].
• The dataset provides new insights on the role of structured packing in the use of wet scrubber for FDG processes. It can be effectively adopted in future works as a comparison term for the development of new and tuned packings for similar FGD processes.
• The dataset provides a matrix of experimental results that can be used for to assess the relations among the fundamental parameters of absorption processes using structured packing.
• These data show the role that the alkalinity of water (used as absorption liquid) plays in the FGD processes [5,6].
• The additional value of these data also relies in the possibility of applying the knowledge achieved so far in the treatment of other gas pollutants, *e.g.* NO_x_, CO_2_, CO, NH_3_ and H_2_S.

## 1 Data Description

The experimental data were acquired using a pilot-scale scrubber equipped with a structured packing (Mellapak 250.X) for the desulphurization of a simulated flue-gas with different scrubbing solutions. The complete dataset provided in this work derives from gas-liquid absorption experimental tests and was collected from two different experimental campaigns. Consequently, it consists of two separate sets of absorption experiments, grouped on the basis of the absorption liquids used.

1. Set of experiments with acidified distilled water and a synthetic seawater with NaOH;
2. Set of experiments with distilled water, a tap water, a synthetic seawater, and seawater with NaOH.

Tables 1–2 show the first set of SO_2_ absorption experiments carried out in a packed column equipped with Mellapak 250.X, with a column diameter *D_C_* = 0.1 m (corresponding to a column section *S_C_* = 0.00785 m^2^) and a packing height *Z_p_* = 0.892 m. The tests were performed at four gas velocities, *u_G_* (1.00, 1.13, 1.27 and 1.41 m⋅s ^−^ ^1^ corresponding to gas flow rates, referred to the column section *S_C_*, equal to 28, 32, 36 and 40 m^3^⋅h ^−^ ^1^) at 25 °C and 1 atm, and variable concentration of SO_2_ in gas-phase, *C_SO_2__* (455 - 650 ppm_v_). The liquid flow rates were 40, 70, 100 and 130 L⋅h ^−^ ^1^ at 25 °C, corresponding to liquid to gas fed ratios (*Q_L_*/*Q_G_*, [L⋅m ^−^ ^3^]) ranging between 1.00 - 4.64 L⋅m ^−^ ^3^.

**Table 1:** Dataset of the absorption experiments using acidified distilled water as scrubbing liquid: pressure drops (ΔP/Z); gas temperatures (T_G_); relative humidity (H_r_) and SO_2_ concentration (C_SO_2__); SO_2_ removal efficiency (η_SO_2__); temperatures (T_L_) and pH of the scrubbing liquid. Data were acquired both before (input data) and after scrubbing tests (output data).

**INPUT DATA**							**OUTPUT DATA**						
u_G_	T_G_	H_r_	C_SO_2__	Q_L_/Q_G_	T_L_	pH	ΔP/Z	T_G_	H_r_	C_SO_2__	η_SO_2__	T_L_	pH
m⋅s ^−^ ^1^	°C	%	ppm_v_	L⋅m ^−^ ^3^	°C	–	mbar⋅m ^−^ ^1^	°C	%	ppm_v_	%	°C	–
1.00	25.0	25.1	513	1.42	25.0	3.00	2.50	24.8	26.8	429	16.37	24.9	2.68
				2.50			2.67	25.0	29.5	376	26.71	24.9	2.69
				3.57			2.83	24.7	33.2	328	36.06	24.9	2.70
				4.64			3.00	24.8	35.2	287	44.05	25.0	2.80
1.13	25.0	25.2	523	1.25	25.0	3.00	2.50	24.7	26.4	438	16.25	24.9	2.58
				2.19			2.67	24.7	28.4	387	26.00	24.8	2.62
				3.12			2.83	25.1	30.5	342	34.61	24.8	2.65
				4.06			3.00	24.8	32.5	308	41.11	25.0	2.68
1.27	25.0	25.3	562	1.11	25.0	3.00	3.50	24.8	25.5	477	15.12	24.9	2.49
				1.94			3.67	24.9	27.7	425	24.38	25.0	2.50
				2.78			3.83	24.7	29.9	381	32.21	24.8	2.51
				3.61			4.08	25.0	31.7	341	39.32	24.9	2.51
1.41	25.0	25.1	530	1.00	25.0	3.00	4.17	24.7	25.3	451	14.91	24.9	2.41
				1.75			4.42	24.8	26.7	406	23.40	24.8	2.44
				2.50			4.67	24.9	28.9	367	30.75	24.8	2.53
				3.25			4.83	24.9	30.2	331	37.55	25.0	2.55

**Table 2:** Dataset of the absorption experiments using a synthetic seawater with NaOH as scrubbing liquid: pressure drops (ΔP/Z); gas temperatures (T_G_); relative humidity (H_r_) and SO_2_ concentration (C_SO_2__); SO_2_ removal efficiency (η_SO_2__); temperatures (T_L_) and pH of the scrubbing liquid. Data were acquired both before (input data) and after scrubbing tests (output data).

**INPUT DATA**							**OUTPUT DATA**						
u_G_	T_G_	H_r_	C_SO_2__	Q_L_/Q_G_	T_L_	pH	ΔP/Z	T_G_	H_r_	C_SO_2__	η_SO_2__	T_L_	pH
m⋅s ^−^ ^1^	°C	%	ppm_v_	L⋅m ^−^ ^3^	°C	–	mbar⋅m ^−^ ^1^	°C	%	ppm_v_	%	°C	–
1.00	25.0	25.0	493	1.42	25.0	9.40	2.50	24.8	26.8	29	94.12	25.0	3.44
			493	2.50			2.67	25.0	29.5	18	96.35	24.9	5.78
			650	3.57			2.83	24.7	33.2	12	98.15	25.1	5.95
			650	4.64			3.00	24.8	35.2	8	98.77	25.0	6.28
1.13	25.0	25.2	548	1.25	25.0	9.40	2.50	24.7	26.4	44	91.97	24.8	3.59
			548	2.19			2.67	24.7	28.4	24	95.62	24.8	5.53
			585	3.12			2.83	25.1	30.5	13	97.78	24.8	5.84
			585	4.06			3.00	24.8	32.5	9	98.46	24.9	6.11
1.27	25.0	25.0	455	1.11	25.0	9.40	3.50	24.8	25.5	43	90.55	25.0	3.13
			455	1.94			3.67	24.9	27.7	22	95.16	25.0	5.44
			555	2.78			3.83	24.7	29.9	14	97.48	24.9	5.84
			626	3.61			4.08	25.0	31.7	10	98.40	24.9	6.03
1.41	25.0	25.1	447	1.00	25.0	9.40	4.17	24.7	25.3	47	89.49	24.9	2.97
			447	1.75			4.42	24.8	26.7	24	94.63	24.8	5.27
			505	2.50			4.67	24.9	28.9	15	97.03	24.8	5.78
			570	3.25			4.83	24.9	30.2	11	98.07	24.8	6.01

Table 3, Table 4, Table 5, Table 6 show the second set of SO_2_ absorption experiments carried out in a packed column equipped with Mellapak 250.X, with a column diameter *D_C_* = 0.1 m (corresponding to a column section *S_C_* = 0.00785 m^2^) and a packing height *Z_p_* = 0.892 m. The tests were performed at a constant gas velocity, *u_G_* (1.13 m⋅s ^−^ ^1^ corresponding to gas flow rate, referred to the column section *S_C_*, equal to 32 m^3^⋅h ^−^ ^1^) at 25 °C and 1 atm, with different SO_2_ fed concentrations, *C_SO_2__* (from 500 to 2000 ppm_v_) and different inlet gas temperatures (25, 40 and 60 °C). The liquid flow rates were 40, 70, 100 and 130 L⋅h ^−^ ^1^ at 25 °C, corresponding to liquid to gas fed ratios (*Q_L_*/*Q_G_*, [L⋅m ^−^ ^3^]) ranging between 1.25 - 4.06 L⋅m ^−^ ^3^.

**Table 3:** Dataset of the absorption experiments using a distilled water as scrubbing liquid: pressure drops (ΔP/Z); gas temperatures (T_G_); relative humidity (H_r_) and SO_2_ concentration (C_SO_2__); SO_2_ removal efficiency (η_SO_2__); temperatures (T_L_) and pH of the scrubbing liquid. Data were acquired both before (input data) and after scrubbing tests (output data).

**INPUT DATA**							**OUTPUT DATA**						
u_G_	T_G_	H_r_	C_SO_2__	Q_L_/Q_G_	T_L_	pH	ΔP/Z	T_G_	H_r_	C_SO_2__	η_SO_2__	T_L_	pH
m⋅s ^−^ ^1^	°C	%	ppm_v_	L⋅m ^−^ ^3^	°C	–	mbar⋅m ^−^ ^1^	°C	%	ppm_v_	%	°C	–
1.13	25.0	25.2	500	1.25	25.0	6.00	2.52	24.8	27.7	400	20.00	25.0	2.30
				2.19			2.64	25.1	29.7	330	34.00	24.9	2.42
				3.12			2.80	24.7	33.1	277	44.60	25.1	2.49
				4.06			3.05	24.9	35.8	246	50.80	25.0	2.61
1.13	25.0	25.1	1000	1.25	25.0	6.00	2.51	24.7	27.2	826	17.40	25.0	2.10
				2.19			2.63	25.0	29.3	724	27.60	25.2	2.24
				3.12			2.78	24.9	33.3	650	35.00	25.3	2.32
				4.06			3.02	25.0	35.4	578	42.20	25.1	2.53
1.13	25.0	24.8	2000	1.25	25.0	6.00	2.59	24.7	27.4	1700	15.00	25.0	2.04
				2.19			2.63	25.2	28.5	1550	22.50	24.9	2.11
				3.12			2.88	24.8	32.4	1427	28.65	25.1	2.19
				4.06			3.09	24.8	34.8	1311	34.45	25.0	2.35
1.13	40.0	18.2	500	1.25	25.0	6.00	2.45	28.9	37.4	405	19.00	25.0	2.25
				2.19			2.66	28.2	39.5	342	31.60	25.2	2.48
				3.12			2.88	27.7	42.7	287	42.60	25.3	2.54
				4.06			3.07	27.4	45.3	252	49.60	25.1	2.62
1.13	40.0	18.1	1000	1.25	25.0	6.00	2.52	28.7	37.4	833	16.70	25.1	2.26
				2.19			2.65	28.5	38.9	730	27.00	25.2	2.37
				3.12			2.83	27.4	43.5	665	33.50	25.1	2.38
				4.06			3.02	27.2	46.5	583	41.70	25.0	2.63
1.13	40.0	18.0	2000	1.25	25.0	6.00	2.54	27.8	38.4	1731	13.45	25.0	2.04
				2.19			2.69	27.5	39.5	1560	22.00	24.9	2.11
				3.12			2.85	27.3	42.4	1416	29.20	25.1	2.19
				4.06			3.04	27.0	47.3	1320	34.00	25.0	2.35
1.13	60.0	13.3	500	1.25	25.0	6.00	2.53	33.5	48.4	407	18.60	27.9	2.46
				2.19			2.65	32.2	51.2	336	32.80	27.6	2.45
				3.12			2.87	31.5	53.6	280	44.00	27.4	2.55
				4.06			3.11	30.1	56.5	249	50.20	27.3	2.63
1.13	60.0	13.1	1000	1.25	25.0	6.00	2.43	33.8	48.9	833	16.70	28.0	2.25
				2.19			2.60	32.4	52.1	727	27.30	28.1	2.30
				3.12			2.88	31.3	54.3	654	34.60	27.7	2.35
				4.06			3.06	30.7	56.8	583	41.70	27.5	2.63
1.13	60.0	12.8	2000	1.25	25.0	6.00	2.54	33.8	47.9	1709	14.55	28.2	2.15
				2.19			2.64	32.1	53.4	1559	22.05	27.9	2.24
				3.12			2.83	31.4	55.4	1450	27.50	27.6	2.24
				4.06			3.05	30.3	56.9	1315	34.25	27.5	2.42

**Table 4:** Dataset of the absorption experiments using a tap water as scrubbing liquid: pressure drops (ΔP/Z); gas temperatures (T_G_); relative humidity (H_r_) and SO_2_ concentration (C_SO_2__); SO_2_ removal efficiency (η_SO_2__); temperatures (T_L_) and pH of the scrubbing liquid. Data were acquired both before (input data) and after scrubbing tests (output data).

**INPUT DATA**							**OUTPUT DATA**						
u_G_	T_G_	H_r_	C_SO_2__	Q_L_/Q_G_	T_L_	pH	ΔP/Z	T_G_	H_r_	C_SO_2__	η_SO_2__	T_L_	pH
m⋅s ^−^ ^1^	°C	%	ppm_v_	L⋅m ^−^ ^3^	°C	–	mbar⋅m ^−^ ^1^	°C	%	ppm_v_	%	°C	–
1.13	25.0	25.2	500	1.25	25.0	7.60	2.51	24.9	27.9	234	53.20	25.1	2.65
				2.19			2.62	25.1	29.5	101	79.80	24.9	4.95
				3.12			2.84	24.8	33.5	36	92.80	25.0	6.02
				4.06			3.03	24.9	35.6	22	95.60	25.0	6.45
1.13	25.0	25.1	1000	1.25	25.0	7.60	2.52	24.8	27.1	670	33.00	25.0	2.34
				2.19			2.66	25.1	29.2	465	53.50	25.1	2.65
				3.12			2.75	24.8	33.8	286	71.40	25.2	3.04
				4.06			3.00	25.0	36.2	156	84.40	25.1	4.02
1.13	25.0	24.8	2000	1.25	25.0	7.60	2.59	24.8	27.3	1608	19.60	25.2	1.98
				2.19			2.63	25.2	28.8	1340	33.00	24.8	2.28
				3.12			2.85	24.9	32.5	1036	48.20	25.0	2.47
				4.06			3.03	25.0	35.2	820	59.00	25.0	2.61
1.13	40.0	18.2	500	1.25	25.0	7.60	2.42	28.9	37.5	225	55.00	25.0	2.90
				2.19			2.65	28.3	39.4	92	81.60	25.3	5.40
				3.12			2.82	27.5	43.1	37	92.60	25.2	6.15
				4.06			3.04	27.5	45.3	23	95.40	25.1	6.50
1.13	40.0	18.1	1000	1.25	25.0	7.60	2.54	28.6	37.3	695	30.50	25.0	2.40
				2.19			2.65	28.4	39.3	496	50.40	25.0	2.90
				3.12			2.85	27.4	43.1	285	71.50	25.1	3.15
				4.06			3.04	27.3	46.4	165	83.50	25.0	4.70
1.13	40.0	17.9	2000	1.25	25.0	7.60	2.53	28.6	38.5	1608	19.60	25.2	2.00
				2.19			2.66	28.7	39.5	1340	33.00	24.9	2.45
				3.12			2.88	27.4	42.6	1036	48.20	25.1	2.50
				4.06			3.03	27.1	47.1	820	59.00	24.9	2.65
1.13	60.0	13.2	500	1.25	25.0	7.60	2.53	33.6	48.3	407	53.00	27.7	2.78
				2.19			2.63	32.3	51.0	336	78.00	27.5	5.20
				3.12			2.86	31.4	53.3	280	91.00	27.4	6.10
				4.06			3.10	30.3	56.0	249	94.60	27.4	6.45
1.13	60.0	13.0	1000	1.25	25.0	7.60	2.45	33.8	49.2	833	30.50	28.3	2.40
				2.19			2.61	32.6	52.2	727	50.20	28.1	2.70
				3.12			2.89	31.5	54.4	654	68.60	27.9	3.12
				4.06			3.04	30.8	57.0	583	81.80	27.7	4.20
1.13	60.0	12.9	2000	1.25	25.0	7.60	2.53	33.8	48.0	1709	18.50	28.1	2.10
				2.19			2.63	32.2	53.6	1559	32.25	27.8	2.30
				3.12			2.85	31.5	55.4	1450	47.50	27.6	2.40
				4.06			3.07	30.4	56.7	1315	58.35	27.6	2.65

**Table 5:** Dataset of the absorption experiments using a synthetic seawater solution as a scrubbing liquid: pressure drops (ΔP/Z); gas temperatures (T_G_); relative humidity (H_r_) and SO_2_ concentration (C_SO_2__); SO_2_ removal efficiency (η_SO_2__); temperatures (T_L_) and pH of the scrubbing liquid. Data were acquired both before (input data) and after scrubbing tests (output data).

**INPUT DATA**							**OUTPUT DATA**						
u_G_	T_G_	H_r_	C_SO_2__	Q_L_/Q_G_	T_L_	pH	ΔP/Z	T_G_	H_r_	C_SO_2__	η_SO_2__	T_L_	pH
m⋅s ^−^ ^1^	°C	%	ppm_v_	L⋅m ^−^ ^3^	°C	–	mbar⋅m ^−^ ^1^	°C	%	ppm_v_	%	°C	–
1.13	25.0	25.2	500	1.25	25.0	8.20	2.50	24.9	27.2	149	70.20	25.1	3.55
				2.19			2.62	25.0	29.5	53	89.40	24.9	5.35
				3.12			2.83	24.8	29.9	17	96.60	25.0	6.03
				4.06			3.02	24.9	35.6	7	98.60	25.0	6.32
1.13	25.0	25.1	1000	1.25	25.0	8.20	2.53	24.9	27.1	550	45.00	25.1	2.58
				2.19			2.62	25.1	29.6	240	76.00	25.2	2.85
				3.12			2.75	24.9	33.4	83	91.70	25.3	3.45
				4.06			3.05	25.0	35.5	30	97.00	25.0	5.12
1.13	25.0	24.8	2000	1.25	25.0	8.20	2.52	24.8	27.7	1430	28.50	25.0	2.12
				2.19			2.62	25.1	28.5	1030	48.50	24.9	2.23
				3.12			2.85	25.0	32.4	664	66.80	25.1	2.29
				4.06			3.07	24.8	34.3	432	78.40	25.1	2.36
1.13	40.0	18.3	500	1.25	25.0	8.20	2.44	28.7	37.2	155	69.00	25.0	3.85
				2.19			2.61	28.1	39.3	55	89.00	25.0	5.40
				3.12			2.86	27.5	42.5	20	96.00	25.0	6.20
				4.06			3.09	27.3	45.6	8	98.40	25.1	6.50
1.13	40.0	18.1	1000	1.25	25.0	8.20	2.50	28.8	37.3	560	44.00	25.1	2.70
				2.19			2.64	28.4	39.1	245	75.50	25.0	2.85
				3.12			2.82	27.5	43.6	91	90.90	25.1	3.40
				4.06			3.01	27.4	46.7	33	96.70	25.1	5.25
1.13	40.0	18.1	2000	1.25	25.0	8.20	2.55	28.5	38.5	1444	27.80	25.0	2.30
				2.19			2.70	28.1	39.6	1044	47.80	25.1	2.45
				3.12			2.85	27.4	42.5	678	66.10	25.0	2.55
				4.06			3.03	27.2	47.5	440	78.00	24.9	2.78
1.13	60.0	13.1	500	1.25	25.0	8.20	2.54	33.4	48.5	142	71.60	27.7	3.60
				2.19			2.66	32.2	51.3	56	88.80	27.5	5.60
				3.12			2.88	31.4	53.5	21	95.80	27.4	6.10
				4.06			3.06	30.0	56.0	9	98.20	27.2	6.35
1.13	60.0	13.0	1000	1.25	25.0	8.20	2.45	33.6	49.3	560	44.00	28.3	2.60
				2.19			2.66	32.5	52.4	262	73.80	28.1	2.90
				3.12			2.83	31.0	54.0	85	91.50	27.8	4.25
				4.06			3.05	30.6	57.2	32	96.80	27.6	5.20
1.13	60.0	12.9	2000	1.25	25.0	8.20	2.55	33.7	47.7	1445	27.75	28.2	2.20
				2.19			2.68	32.0	53.3	1043	47.85	27.0	2.40
				3.12			2.87	31.5	55.1	673	66.35	27.5	2.30
				4.06			3.09	30.4	57.3	445	77.75	27.3	2.65

**Table 6:** Dataset of the absorption experiments using a synthetic seawater solution with 200 mg⋅L ^−^ ^1^ of NaOH addition as scrubbing liquid: pressure drops (ΔP/Z); gas temperatures (T_G_); relative humidity (H_r_) and SO_2_ concentration (C_SO_2__); SO_2_ removal efficiency (η_SO_2__); temperatures (T_L_) and pH of the scrubbing liquid. Data were acquired both before (input data) and after scrubbing tests (output data).

**INPUT DATA**							**OUTPUT DATA**						
u_G_	T_G_	H_r_	C_SO_2__	Q_L_/Q_G_	T_L_	pH	ΔP/Z	T_G_	H_r_	C_SO_2__	η_SO_2__	T_L_	pH
m⋅s ^−^ ^1^	°C	%	ppm_v_	L⋅m ^−^ ^3^	°C	–	mbar⋅m ^−^ ^1^	°C	%	ppm_v_	%	°C	–
1.13	25.0	25.2	500	1.25	25.0	9.40	2.52	24.8	27.5	111	77.80	25.0	4.86
				2.19			2.62	25.0	29.1	35	93.00	24.9	6.11
				3.12			2.85	24.9	30.1	11	97.80	25.1	6.82
				4.06			3.00	25.0	35.8	3	99.40	25.0	7.76
1.13	25.0	25.0	1000	1.25	25.0	9.40	2.54	24.9	27.0	430	57.00	25.0	2.54
				2.19			2.62	25.0	29.2	194	80.60	25.0	4.22
				3.12			2.78	25.1	33.7	75	92.50	25.1	5.45
				4.06			3.10	25.0	35.9	26	97.40	25.0	5.98
1.13	25.0	24.8	2000	1.25	25.0	9.40	2.51	24.9	28.0	1300	35.00	25.0	2.19
				2.19			2.65	25.0	28.6	835	58.25	25.0	2.67
				3.12			2.86	25.0	32.5	515	74.25	25.1	2.92
				4.06			3.05	24.9	34.7	290	85.50	25.1	3.16
1.13	40.0	18.3	500	1.25	25.0	9.40	2.45	28.9	37.1	115	77.00	24.8	4.95
				2.19			2.66	28.2	39.0	40	92.00	25.1	6.15
				3.12			2.89	27.4	42.7	13	97.40	25.0	6.85
				4.06			3.08	27.5	45.9	3	99.40	25.1	7.98
1.13	40.0	18.1	1000	1.25	25.0	9.40	2.54	28.9	37.1	445	55.50	25.0	2.70
				2.19			2.65	28.5	38.9	204	79.60	25.0	4.60
				3.12			2.87	27.5	43.5	80	92.00	25.1	5.45
				4.06			3.05	27.3	46.4	28	97.20	25.0	6.15
1.13	40.0	18.0	2000	1.25	25.0	9.40	2.56	28.4	38.5	1320	34.00	25.0	2.30
				2.19			2.70	28.0	39.9	844	57.80	25.0	2.55
				3.12			2.86	27.5	42.4	525	73.75	25.0	3.00
				4.06			3.05	27.1	47.8	300	85.00	24.9	3.45
1.13	60.0	13.2	500	1.25	25.0	9.40	2.55	33.5	48.4	105	79.00	27.8	5.00
				2.19			2.60	32.4	51.0	41	91.80	27.6	6.20
				3.12			2.87	31.4	53.4	15	97.00	27.5	6.80
				4.06			3.05	30.3	56.2	5	99.00	27.2	8.20
1.13	60.0	13.0	1000	1.25	25.0	9.40	2.46	33.5	49.5	444	55.60	28.4	2.60
				2.19			2.67	32.5	52.3	190	81.00	28.1	4.10
				3.12			2.85	31.2	54.1	73	92.70	27.8	5.50
				4.06			3.04	30.5	57.0	23	97.70	27.5	6.10
1.13	60.0	12.8	2000	1.25	25.0	9.40	2.52	33.5	47.9	1321	33.95	28.1	2.20
				2.19			2.66	32.1	53.4	865	56.75	27.1	2.35
				3.12			2.88	31.4	55.0	520	74.00	27.5	2.80
				4.06			3.10	30.6	57.5	298	85.10	27.0	3.80

The data contains both input and output values of the fundamental parameters of a wet scrubbing FDG process using different gas velocities, liquid to gas fed ratios, gas temperatures and scrubbing liquids, at different pH values.

Starting from input and output SO_2_ concentrations, the removal efficiency (*η_SO_2__*) reported in Table 1, Table 2, Table 3, Table 4, Table 5, Table 6 was determined from Eq. (1):

(1) $$\eta_{SO_{2}}=\frac{C_{SO_{2(g)}}^{IN}-C_{SO_{2(g)}}^{OUT}}{C_{SO_{2(g)}}^{IN}}\cdot 100$$

## 2 Experimental Design, Materials and Methods

### 2.1 Materials

The simulated flue-gas was prepared by mixing SO_2_ at 2% v/v in N_2_ stored in high-pressure cylinders (supplied by Rivoira Gas Srl, Italy) with compressed air at technical grade. Scrubbing experiments were carried out with different scrubbing liquids, listed in the following:

- Acidified distilled water (pH = 3.0, adding 98 mg⋅L ^−^ ^1^ of HCl aqueous solution to distilled water);
- Pure distilled water (pH = 6.0);
- Tap water (pH = 7.6);
- Synthetic seawater solution (pH = 8.2, in the following referred as seawater) obtained by adding 33 g⋅L ^−^ ^1^ of NaCl, 4.14 g⋅L ^−^ ^1^ of Na_2_SO_4_, 0.16 g⋅L ^−^ ^1^ of NaHCO_3_ and 0.03 g⋅L ^−^ ^1^ of Na_2_CO_3_ to the tap water;
- Basic aqueous solution (pH = 9.4, adding 200 mg⋅L ^−^ ^1^ of NaOH to seawater).

The chemicals used for acid and basic aqueous solutions were hydrochloric acid solution (37% w/w) and sodium hydroxide in pellets (99.99% w/w), purchased from VWR International Chemicals (Italy) as AR grade. The tap water composition in terms of the main ions present is reported in Table 7:

**Table 7:** Main ion concentrations in the tap water. The analytical determination was performed by ionic chromatography (Metrohm AG, 883 Basic IC PLUS).

**Solution**	pH-	Cl^−^ g⋅L ^−^ ^1^	SO_4_^2–^ g⋅L ^−^ ^1^	HCO_3_^–^ g⋅L ^−^ ^1^	CO_3_^2–^ mg⋅L ^−^ ^1^	NO_3_^−^ mg⋅L ^−^ ^1^	Na^+^ g⋅L ^−^ ^1^	Mg^2+^ mg⋅L ^−^ ^1^	*K*^+^ mg⋅L ^−^ ^1^	Ca^2+^ g⋅L ^−^ ^1^
Tap water	7.60	0.01	0.01	0.53	< 1	4.21	0.03	26.18	2.34	0.11

### 2.2 Experimental set-up

**Fig. 1:**
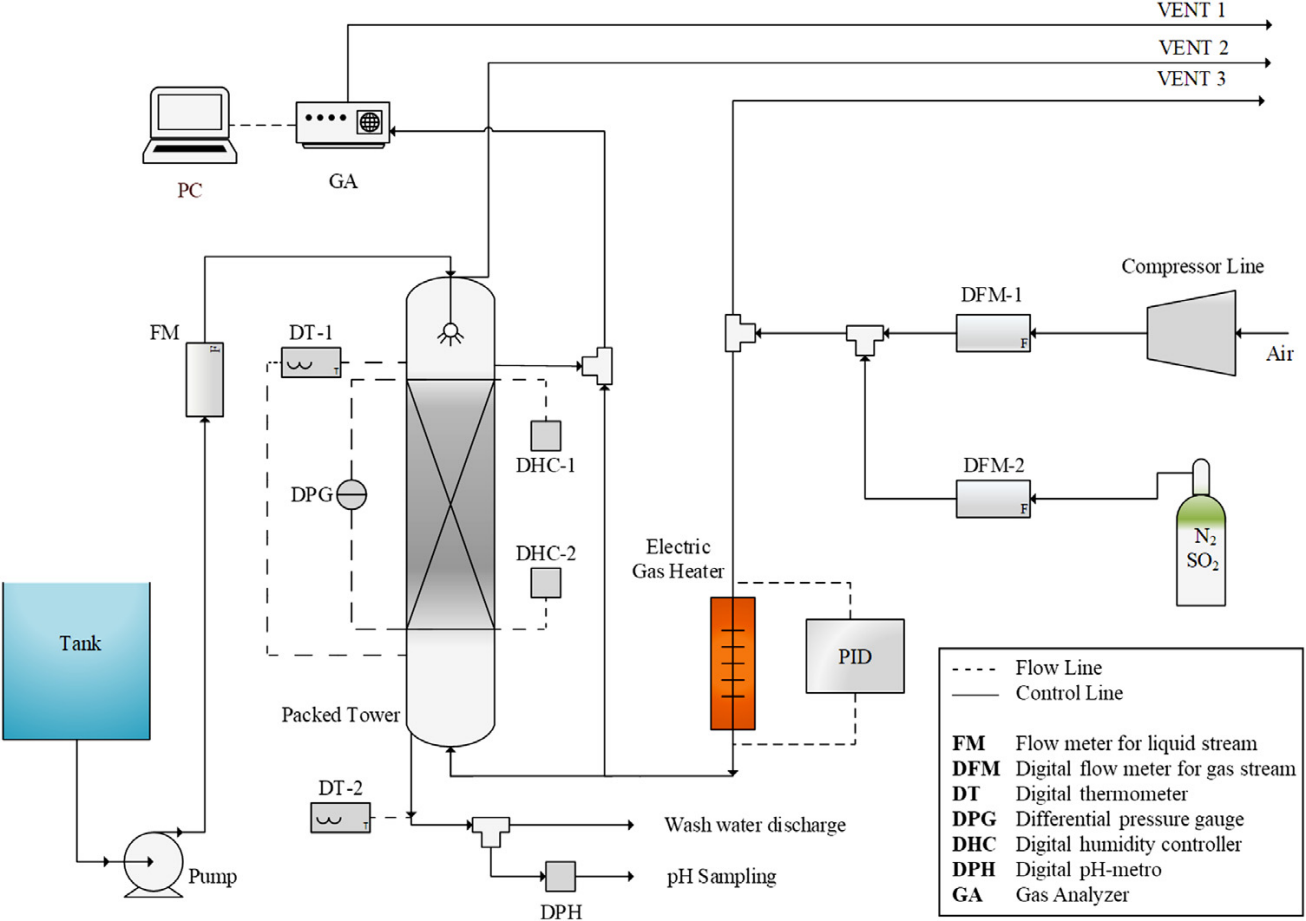
Flowsheet of the experimental set-up including all column equipment and measuring and analysis instruments [2], [3],5].

The flowsheet of the experimental set-up, inclusive of all the column equipment and measuring and analytical instruments, is shown in Fig. 1.

**Fig. 2:**
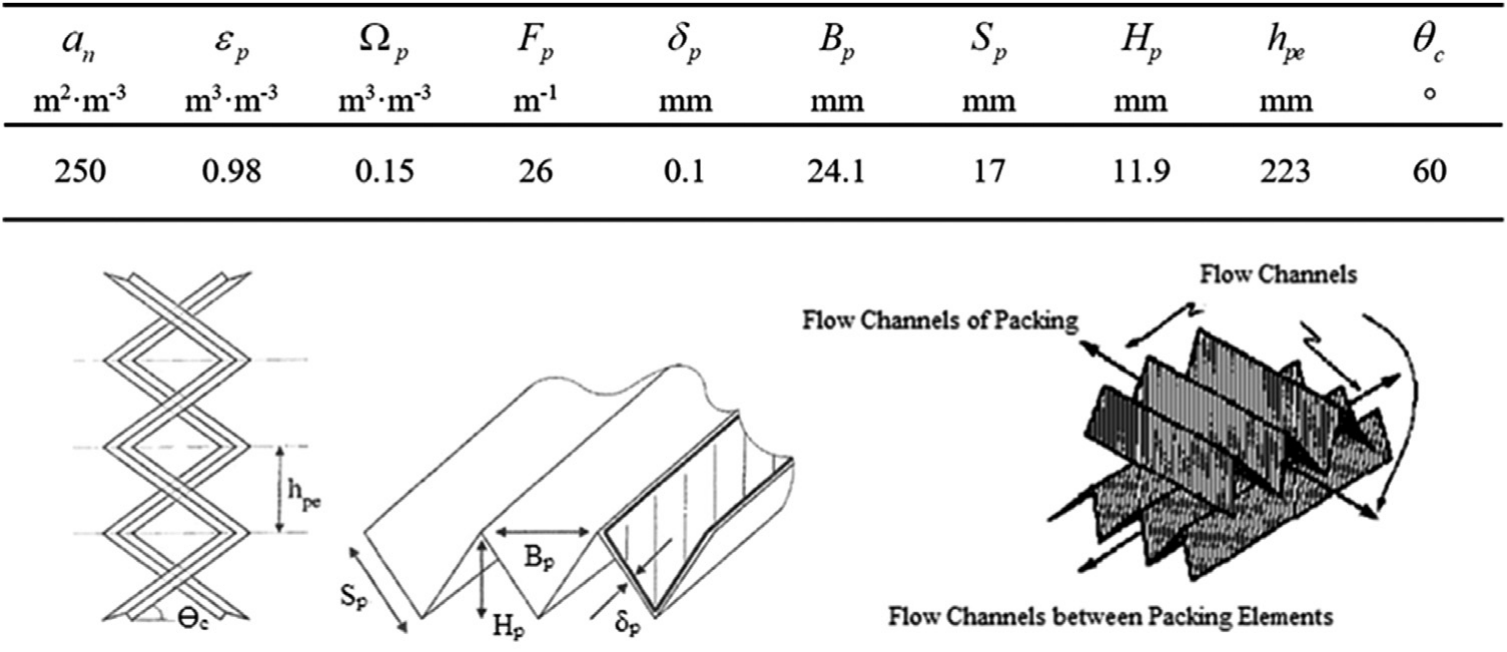
Geometric characteristics of Mellapak 250.X provided by Sulzer Chemtech (upper figure) and structured packing details with some characteristic dimension parameters (lower figure).

SO_2_ absorption experiments were performed in a Plexiglas column (column diameter, *D_C_* = 0.1 m; total column height, *Z* = 1.6 m) operated in the range of temperature 25 - 60 °C and 1 atm. A structured packing with a total packing height *Z_C_* = 0.892 m (Mellapak 250.X, provided by Sulzer Chemtech) was used as filling material. Mellapak 250.X modules are made in Hastelloy C-22 alloy, which was selected to prevent acid corrosion effects during SO_2_ absorption. The geometric characteristics of the Mellapak 250.X packing are reported in Fig. 2. Further details are reported in Flagiello et al. [2,5].

In details: *a_n_* [m^2^⋅m ^−^ ^3^] is the nominal surface area of packing; *ε_p_* [m^3^⋅m ^−^ ^3^] is the void fraction of the packing; *Ω_p_* [m^3^⋅m ^−^ ^3^] is the fraction of packing surface area occupied by holes; *F_p_* [*m* ^−^ ^1^] is the packing factor; *δ_p_* [mm] is the packing thickness; *B_p_* [mm] is the base width of a packing corrugation; *S_p_* [mm] is the slant height of a packing corrugation; *H_p_* [mm] is the peak height of a packing corrugation; *h_pe_* [mm] is the height of a single packing module; *θ_c_* [°] is the corrugation packing angle or inclination angle.

The experimental apparatus can be divided into dedicated sections:

- Gas feed section (gas mixture cylinder, compressor and electric gas heater exchanger);
- Liquid feed section (liquid tank and pump);
- Packed column (structured packing, gas diffuser, gas distributor, spray nozzle and demister);
- Analytical section (SO_2_ gas analyser and digital pH-meter).

A complete regulation system of all the fluid dynamic parameters is also present, consisting of flow meters and temperature, pressure and relative humidity probes.

All the experimental runs were made with a simulated flue gas obtained by mixing SO_2_ in N_2_, available from a cylinder, with air supplied by a compressor. The feeding gas section was managed via SMC Corporation digital flow meters (a PFMB7202-F06-F model able to measure up to 2000 L⋅min^−1^ for air and a PFMB7201S-F02-DWSA model up to 100 L⋅min^−1^ for gas mixtures in cylinders). The simulated flue-gas had an inlet relative humidity in the range 13 - 25%, deriving from air, in the operating gas temperature range between 25 - 60 °C. The model flue-gas temperature was set using an aluminum tubular electric gas heater (i.d. 36 mm and length 250 mm) supplied by Megaris srl (total power of 1 kW). The heat exchanger was connected to a PID controller (Omron E5CB with K-type thermocouples) for temperature control.

The scrubbing liquid was fed at the top of the column, in counter-current flow to the gas, by a Grundfos Lenntech centrifugal pump (CR 3–8 A-A-A-EHQQE model, with total power 0.75 kW) and controlled with a Cryotek Engineering flow meter (D2 model). The pH and temperature of the feeding liquid were measured with a HOBO® digital pH-meter (PCE-228 model, with accuracy of ±0.01 of pH) and a WINGONEER™ mini digital LCD thermometer (with accuracy of ±0.1), respectively. The chemical composition of the tap water used for some of the investigated absorbing liquids was determined by ionic chromatography method, using a Metrohm, AG 883 Basic IC PLUS (see Table 7)

The liquid was fed in the column by a PNR® full cone nozzle (DAM 1212 B31 model) with a complete opening of the liquid jet of 45° The nozzle was positioned on the top of the column, at a defined distance from the packing (35 mm) so to allow a uniform wetting of the packing surface from the top. A 90 mm height plastic foam demister was put at 15 mm from the nozzle at the top of the column to block the entrained liquid drops.

The gas pressure at the top and the bottom of the column was measured by a differential pressure gage (FLUKE Corporation, Air Flow Meter 922 model with accuracy of ±0.1 mm_H2O_). A HOBO® four-channels digital thermometer (PCE T-390 model with accuracy ±0.1 °C) was used for gas temperature measure via K-type thermocouples placed at different column levels, in order to obtain the temperature profile along the column. Finally, the relative humidity content in the gas stream was measured with a HOBO® onset digital humidity controller, UX100–23 model (with accuracy of ±0.1% of relative humidity), at both the inlet/outlet and along the column.

Absorption tests were carried out by feeding the simulated flue-gas stream to the column at the desired flow rate (*Q_G_*, [m^3^⋅h ^−^ ^1^]) or gas velocity (*u_G_*, [m⋅s ^−^ ^1^]), temperature (*T_G_*, [°C]), relative humidity (*H_r_*) and SO_2_ concentration (*C_SO_2__* [ppm_v_]), which was checked by the gas analyzer before the liquid feeding. The scrubbing liquid stream was fed in counter-current flow to the gas flow at the desired flow rate (*Q_L_*, [L⋅h ^−^ ^1^]) and temperature (*T_L_*, [°C]). The SO_2_ gas concentration was monitored and recorded up to a steady state, which takes a characteristic time to reach, dependent on the scrubber fluid-dynamics and its operating conditions.

The concentration of SO_2_ in the gas stream was measured via the ABB O2020® Advanced optima process gas analyzer (range of detection from 0 to 5000 ppm_v_, with an accuracy of ±5 ppm_v_). On the gas line leading to the analytical cell, a gas sampling system was installed upstream to the gas analyzer, consisting of a KNF diaphragm pump (NMP 830 HP model), a Key Instruments flow meter (2500 Series, up to 1 L⋅min^−1^) and a Bühler Technologies gas quencher (TC-Standard Series). The experimental SO_2_ removal efficiency (*η_SO_2__*) was calculated by comparing the input and output SO_2_ concentration, as by Eq. (1).

The wash water was collected at the bottom of the column and sent to a sampling point for further analysis: pH value by HOBO® digital pH-meter (PCE-228 model) and temperature by WINGONEER™ mini digital LCD thermometer were recorded both before (input) and after (output) scrubbing operation.

## CRediT Author Statement

Domenico Flagiello: Conceptualization; Investigation; Resources; Methodology; Data Curation; Writing - Original Draft; Corresponding Author.

Alessandro Erto: Conceptualization; Writing - Review & Editing; Visualization.

Amedeo Lancia: Visualization; Supervision; Project administration; Funding acquisition.

Francesco Di Natale: Conceptualization; Writing - Review & Editing; Visualization.

## Declaration of Competing Interest

The authors declare that they have no known competing financial interests or personal relationships which have, or could be perceived to have, influenced the work reported in this article.
